# *Loranthus tanakae* Franch. & Sav. Suppresses Inflammatory Response in Cigarette Smoke Condensate Exposed Bronchial Epithelial Cells and Mice

**DOI:** 10.3390/antiox11101885

**Published:** 2022-09-23

**Authors:** So-Won Park, A Yeong Lee, Je-Oh Lim, Se-Jin Lee, Woong-Il Kim, Yea-Gin Yang, Bohye Kim, Joong-Sun Kim, Sung-Wook Chae, Kun Na, Yun-Soo Seo, In-Sik Shin

**Affiliations:** 1 College of Veterinary Medicine and BK21 FOUR Program, Chonnam National University, 77 Yongbong-ro, Buk-gu, Gwangju 61186, Korea; 2 Herbal Medicine Resources Research Center, Korea Institute of Oriental Medicine, 177 Geonjae-ro, Naju-si 58245, Korea; 3 Department of Biotechnology, The Catholic University of Korea, 43 Jibong-ro, Wonmi-gu, Bucheon-si 14662, Korea; 4 Department of BioMedical-Chemical Engineering, The Catholic University of Korea, 43 Jibong-ro, Wonmi-gu, Bucheon-si 14662, Korea; 5 KM Convergence Research Division, Korea Institute of Oriental Medicine, 1672 Yuseong-daero, Yuseong-gu, Daejeon 34054, Korea; 6 Center for Companion Animal New Drug Development, Jeonbuk Branch, Korea Institute of Toxicology (KIT), 30 Baekhak1-gil, Jeongeup-si 56212, Korea

**Keywords:** *Loranthus tanakae* Franch. & Sav., cigarette smoke condensate, chronic obstructive pulmonary disease, NF-κB, Nrf2

## Abstract

*Loranthus tanakae* Franch. & Sav. found in China, Japan, and Korea is traditionally used for managing arthritis and respiratory diseases. In this study, we analyzed the components of *L. tanakae* 70% ethanol extract (LTE) and investigated the therapeutic effects of LTE on pulmonary inflammation using cells exposed to cigarette smoke condensate (CSC) and lipopolysaccharide (LPS) in vitro and in vivo in mice and performed a network analysis between components and genes based on a public database. We detected quercitrin, afzelin, rhamnetin 3-rhamnoside, and rhamnocitrin 3-rhamnoside in LTE, which induced a significant reduction in inflammatory mediators including interleukin (IL)-1β, IL-6, tumor necrosis factor (TNF)-α and inflammatory cells in CSC exposed H292 cells and in mice, accompanied by a reduction in inflammatory cell infiltration into lung tissue. In addition, LTE increased translocation into the nuclei of nuclear factor erythroid-2-related factor 2 (Nrf2). By contrast, the activation of nuclear factor (NF)-κB, induced by CSC exposure, decreased after LTE application. These results were consistent with the network pharmacological analysis. In conclusion, LTE effectively attenuated pulmonary inflammation caused by CSC+LPS exposure, which was closely involved in the enhancement of Nrf2 expression and suppression of NF-κB activation. Therefore, LTE may be a potential treatment option for pulmonary inflammatory diseases including chronic obstructive pulmonary disease (COPD).

## 1. Introduction

Cigarette smoke (CS) is associated with the progression of numerous diseases and is regarded as a primary cause in the pathogenesis of chronic obstructive pulmonary disease (CODP) [1]. CS contains several harmful substances, including chemicals, heavy metals, oxidant radicals, and carcinogens, which increase the risk of developing various diseases [2]. Exposure to CS induces the generation of cytokines, reactive oxygen species (ROS), and chemokines, accompanied by the activation of inflammatory signaling [3]. These events can substantially elevate the accumulation of inflammatory cells into pulmonary tissue, eventually leading to pathophysiological alterations, such as the destruction of normal lung tissue, mucus secretion and fibrosis, resulting in the loss of lung function [4]. As it is difficult to quit smoking due to the addictive nature of cigarettes, COPD eventually requires treatment [5]. If various therapeutic agents used, it could be possible to effectively treat the symptoms of COPD [6]. Many therapeutic agents for treating COPD have been developed, focusing on the suppression of inflammatory responses in the respiratory tract [7]. However, currently recommended therapeutics for treating COPD are limited in application because of their adverse effects, including immunosuppression, depression, tolerance and low efficacy [8]. Therefore, it is necessary to develop therapeutic agents with low toxicity and high efficacy.

*Loranthus tanakae* Franch. & Sav. is distributed in China, Japan and Korea, is used as a traditional herbal remedy to control tumors, arthritis, and respiratory disorders [9,10]. It is traditionally consumed as a tea after hot water extraction or after soaking in alcohol. In previous studies, *Loranthus* species have several pharmacological features, including anti-inflammatory, antimicrobial, antioxidant, and antitumor effects, associated with the components found in *L. tanakae*, such as rhamnetin 3-O-α-rhamnoside, rhamnocitrin 3-O-α-rhamnoside, quercitrin, and afzelin [11,12]. However, the effects of *L. tanakae* against COPD have not been explored to date. In predicting the effect of medicinal plants, it is important to the predict the correlation between ingredient and genes [13]. The identification of gene networks controlled by ingredients is one way to garner information about how ingredients exhibit their specific therapeutic efficacy because a characteristic of medicinal plants is that they are the multi-ingredient, multi-targets, and multi-biological activities [14,15]. A good explanation for this role is the network pharmacology approach by Hopkins in 2008 [16]. Considering previous studies, we hypothesized that *L. tanakae* would have the potential to treat COPD.

Therefore, we evaluated the therapeutic effects of *L. tanakae* on COPD using cigarette smoke condensate (CSC) exposed cells and in CSC+LPS exposed mice to explore new applications for the herbal medicine. In addition, to determine the mechanism of action of *L. tanakae*, we investigated the protein expression related to inflammatory responses and oxidative stress of COPD.

## 2. Materials and Methods

### 2.1. Plant and Instrument

#### 2.1.1. Plant Material

The aerial parts of *L. tanakae* were purchased from a herbalist in Jeongseon, Gangwon-do in Republic of Korea. The plant (voucher specimen No. 2-16-0335) was authenticated by Drs. Sungyu Yang and Byeong Cheol Moon at the Korea Institute of Oriental Medicine (KIOM). After air-drying, the plant was pulverized using a blender (Hanil, Seoul, Republic of Korea) and passed through a 600 μm sieve.

#### 2.1.2. Extraction and Isolation

*L. tanakae* (1.032 kg) was refluxed with 6 L of 70% ethanol for 2 h twice. The extraction filtered through chromatography paper (46 × 57 cm) and removed the solvent in vacuo. The yield of the *L. tanakae* 70% ethanol extract (LTE) was 126.42 g (12.25%, *w*/*w*) and it was stored at −20 °C. Four of the major compounds were isolated using preparative high performance liquid chromatography (HPLC, Prep LC) from the 70% ethanol extract. A 100 mg aliquot of LTE was dissolved in 70% ethanol (1 mL) and filtered through a syringe filter (0.45 μm) before injecting it into the HPLC system. The Prep LC system (Waters, Milford, MA, USA) consisted of a Waters 2545 quaternary gradient module, Waters 2998 photodiode array detector, Waters flexinject, and Waters fraction collector III. Four components were isolated using a Phenomenex Synergi 4μ Fusion-RP 80A (250 × 21.20 mm, Phenomenex Inc., Torrance, CA, USA). The mobile phase was prepared by mixing 0.05% aqueous formic acid (A) and acetonitrile (B), and set in a linear gradient program; 5% A → 30% A for 60 min. The flow rate was 5 mL/min and injected volume was 500 μL at room temperature and detection was conducted at a wavelength of 254 nm. Chemical structures were determined with spectroscopic methods using mass spectroscopy and NMR.

#### 2.1.3. HPLC Analysis

Before HPLC analysis, LTE (23.3 mg) was dissolved in 70% ethanol (4 mL) and filtered through a syringe filter (0.2 μm). The HPLC system (Waters, Milford, MA, USA) consisted of an Acquity QDa detector (Waters), a 2998 PDA detector (Waters), a Separation Module (Waters e2695) and a Micro-splitter (IDEX Health & Science LLC, Oak Habor, WA, USA). Three components in 70% ethanol extracts were explored using the XSelect^TM^ HSS T3 column (5 μm, 4.6 × 250 mm, Waters). The mobile phase was prepared by mixing aqueous formic acid (0.05%) (A), Methanol (B) and formic acid (0.05%) in acetonitrile (C), and a linear gradient program was followed; 90% A (3% B, 7% C) to 70% A (10% B, 20% C) for 0–4 min, 70% A (10% B, 20% C) to 63% A (13% B, 24% C) for 4–9 min, 63% A (13% B, 24% C) to 60% A (15% B, 25% C) for 9–12.5 min, 60% A (15% B, 25% C) to 50% A (20% B, 30% C) for 12.5–23 min, 50% A (20% B, 30% C) to 45% A (23% B, 43% C) for 23–26 min, 45% A (23% B, 43% C) to 30% A (30% B, 40% C) for 26–35 min, 30% A (30% B, 40% C) to 0% A (40% B, 60% C) for 35–45 min, and 0% A isocratic for 45–55 min. The flow rate was 0.8 mL/min, injected volume was 10 μL, and column temperature was adjusted. UV wavelength was monitored from 210 to 400 nm, and three target peaks were detected at 254 nm. QDa conditions were set up as the follows: nitrogen as the carrier gas, positive/negative TIC mode, ESI capillary at 0.80 kV, probe temperature of 600 °C, Con Voltage of 15 V, source temperature of 120 °C, and 210:1 split.

### 2.2. Network Pharmacology Analysis

#### 2.2.1. Small Molecules and Potential Target Genes

Genes related to the four active components were collected from the SwissTargetPrediction database (http://www.swisstargetprediction.ch/, accessed on 22 May 2022) with ‘*Homo sapiens*’ species and probability > 0. In this database, we used the probability originated from our cross-validation analysis to rank the targets and assess the accuracy of the predictions [17]. Potential target genes were searched in GeneCards: Human Gene Database (https://www.genecards.org/. version 5.11, accessed on 18 May 2022), and selected according to the intersection of the genes related to the active small molecules with the COPD-related genes.

#### 2.2.2. Protein–Protein Interaction (PPI)

PPI is the process by which two or more proteins form a complex through non-covalent bonds [18]. Sophisticated network-based tools have been designed to anticipate potential disease genes [19]. PPI analyses were performed with the STITCH database (http://stitch.embl.de/, version 5, accessed on 18 May 2022) with a medium confidence score (≥0.400), and topology of PPI was conducted by Cytoscape version 3.7.2 (https://cytoscape.org/, accessed on 18 May 2022) [19].

#### 2.2.3. Signal Pathway Analysis

Signal pathway analyses were carried out using DAVID informatics Resources (https://david.ncifcrf.gov/, accessed on 27 May 2022) version 6.8 and KEGG: Kyoto Encyclopedia of Genes and Genomes (https://www.genome.jp/kegg/, accessed on 27 May 2022) with *p* > 0.05. The network was visualized using Cytoscape version 3.7.2 (Cytoscap, Boston, MA, USA).

### 2.3. In Vitro Experiment

#### 2.3.1. Cell Viability

A human airway epithelial cells (NCI-H292, American Type Culture Collection (ATCC), Manassas, VA, USA) were maintained in RPMI 1640 added with 10% heat-inactivated fetal bovine serum and antibiotics at 37 °C in a 5% CO_2_ incubator. Cell viability was determined using an EZ-Cytox kit (DAELIL, Seoul, Korea).

#### 2.3.2. Evaluation of Inflammatory Cytokines in H292 Cells

The cells were seeded on 6 well plates (4 × 10^5^ cells/well) for 24 h, then treated with LTE at 12.5, 25, 50 and 100 μg/mL. After incubation for 1 h, the cells were treated with CSC (100 μg/mL) for 24 h. The supernatant of cells was collected and determined the generation of interleukin (IL)-1β and IL-6 using commercial ELISA kit (BD Science, San Diego, CA, USA). The CSC was prepared as previously described [20]. Briefly, the CSC sample was prepared by smoking the cigarettes (research reference cigarette 3R4F, University of Kentucky, Lexington, KY, USA) on the 30-port smoking machine in according to International Organization for Standardization (ISO) 3308 (puff duration 2 s, puff volume 35 mL, puff interval 60 s and no vent blocking). The CSC was collected from generated cigarette smoke at 1 L/min for 5 min using a Whatman Cambridge filter pad (44 mm diameter glass fiber filter, GE Healthcare, Buckinghamshire, UK) and a mini vacuum pump (XR5000, SKC Inc., Covington, GA, USA). The total particulate matter (TPM) on the filter pad was extracted with methanol for 30 min with twist shaker to yield a concentration of 5 mg/mL (TPM mass per methanol volume). The 50 mL tubes containing the aliquots of this TPM (40 mL/tube) were subsequently vaporized in a vacuum dry oven overnight.

#### 2.3.3. Immunofluorescence for Nrf2 and NF-κB in H292 Cells

Double-immunofluorescence was conducted according to previous study [21], using anti- NF-κB (ab32536, 1:100 dilution, Abcam, Cambridge, UK), anti- Nrf2 (ab31163, 1:100 dilution, Abcam) antibodies, and a confocal laser scanning microscope (LMS900, ZEISS, Dresden, Germany).

#### 2.3.4. DPPH Radical Scavenging Activity

1,1-diphenyl-2-picrylhydrazyl (DPPH) and 2,2′-zaino-bis-3-ethylbenzthiazoline-6-sulphonic acid (ABTS) free radical scavenging activity for measurement of antioxidant capacities were determined according to reported [22,23]. DPPH solution dissolved in methanol and 100 μL LTE (62.5, 125, 250, and 500 μg/mL) reacted at 37 °C in incubator for 30 min. The absorbance of 100 μL DPPH radical was measured at 517 nm. For the ABTS assay, radical solution reacted with phosphate was adjusted to a final absorbance of 0.7 ± 0.02 at 734 nm. Gallic acid was used as a positive control in this study. The scavenging rate of DPPH and ABTS of the LTE was calculated according to the following formula:

% scavenging rate = [(Abs_free radical_ − Abs_control_)/Abs_free radical_] × 100

#### 2.3.5. Measurement of ROS Production

To measure ROS production, HeLa cells (3 × 10^5^/well, ATCC) were treated with rhamnetin 3-rhamnoside (5, 10, 50 and 100 μg/mL) and quercitrin (5, 10, 50 and 100 μg/mL). After incubation for 6 h, cells were treated with H_2_O_2_ (1 mM) for 45 min. Additionally, then cells were stained with 2′,7′-dichlorodihydrofluorescein diacetate (10 μM, DCFDA, Sigma-Aldrich, Saint Louis, MO, USA) for 45 min. The images were acquired on a confocal laser scanning microscope (LMS900, ZEISS).

### 2.4. In Vivo Experiment

#### 2.4.1. Animals

Male C57BL/6N mice (6 weeks old, Samtako Co., Osan, Korea) were maintained under standard conditions with food and water ad libitum. The procedure of in vivo was obtained approval from the Institutional Animal Care and Use Committee of the Chonnam National University (CNU IACUC-YB-2021-39).

#### 2.4.2. Procedure of Animal Experiments

To establish COPD model, we used CSC and LPS exposed mouse model. In the development of COPD, besides exposure to CS, bacterial, fungi, virus and air-pollutants are also important risk factors potentially aggravating clinical sign of COPD [24]. In previous report, additional exposure to LPS in cigarette smoke induced experimental model for COPD induces an excessive inflammatory response in the respiratory tract [20,25]. The animals were assigned into 5 groups (*n* = 5) as follows: normal control (PBS intranasal instillation and oral gavage), CSC+LPS (CSC+LPS intranasal instillation and PBS oral gavage), ROF (CSC+LPS intranasal instillation and roflumilast (10 mg/kg of body weight) oral gavage), LTE 50 and 100 (CSC+LPS intranasal instillation and LTE oral gavage (50 and 100 mg/kg of body weight, respectively)). CSC (12.5 mg/kg of body weight) and LPS (0.5 mg/kg of body weight) were intranasally administered to animals under slight anesthesia on days 1, 6 and 13; the animals were sacrificed on day 15. To obtain bronchoalveolar lavage fluids (BALF), the mice were tracheostomized under anesthesia and endotracheal tubes were then inserted. PBS (0.7 mL) was infused into the lungs and withdrawn, and this procedure was repeated once (total volume: 1.4 mL). The inflammatory cell counts of BALF were assessed as previously described [21]. The supernatant of BALF was used for the evaluation of cytokines.

#### 2.4.3. Measurement of Inflammatory Mediators in BALF

The generation of IL-6 and TNF-α in BALF were assessed using commercial ELISA kits (BD Science) according to the manufacturer’s protocols.

#### 2.4.4. Histopathology of Lung Tissue

The left lung tissue was fixed in neutralized formalin and stained with hematoxylin and eosin per standard procedures (thickness of sections: 4 μm) to evaluate pulmonary inflammation. To evaluate the expression NRF-2 and NF-κB on lung tissue, we conducted immunohistochemistry as previously described [19]. The following primary antibodies were used: NF-κB (1:100 dilution, Abcam) and NRF-2 (1:100 dilution, Abcam). Quantitative analysis of pulmonary inflammation, mucus production and protein expression was performed using an image analyzer (IMT i-Solution Inc., Vancouver, BC, Canada).

### 2.5. Statistical Analysis

The data were expressed as the mean ± standard deviation (SD). Statistical significance was determined using an analysis of variance (ANOVA) followed by a multiple comparison test with Dunnet’s adjustment. *p* values < 0.05 were considered significant.

## 3. Results

### 3.1. Isolation of Active Components

LTE was separated using PrepLC with gradient solvent program and isolated four known flavonoids. These molecules were confirmed by comparisons with the literature and commercial standard compounds, as quercitrin (10 mg) [26], afzelin (6 mg) [27], rhamnetin -3-rhamnoside (15 mg) [28], and rhamnocirin 3-rhamnoside (11 mg) [11]. The purity of these single compounds was confirmed by HPLC, and all of them were separated to more than 95%, which is the similar as commercial compounds. The purity of each compound is as follows; 95.82% of quercitrin, 98.98% of afzelin, 98.87% of rhamnoside 3-rhamnoside, and 99.08% of rhamnocitrin 3-rhamnoside (Supplementary Materials Figure S1).

### 3.2. HPLC Analysis of LTE

**Figure 1:**
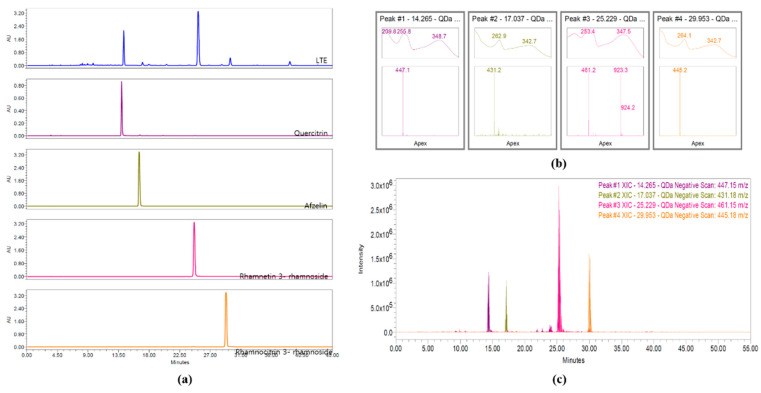
Analysis of ingredients of LTE. (**a**) HPLC chromatogram of LTE and active molecules at 254 nm, (**b**) verification of four main peaks comparing λ_max_, *m*/*z* for [M−H]^−^; quercitrin (purple, λ_max_ = 255.8, *m*/*z* = 447.1), afzelin (olive green, λ_max_ = 262.9, *m*/*z* = 431.2), rhamnetin 3-rhamnoside (pink, λ_max_ = 253.4, *m*/*z* = 461.2), and rhamnocitrin (orange, λ_max_ = 264.1, *m*/*z* = 445.2), and (**c**) overlay of four SIR peaks in LTE; quercitrin (purples; 14.265 min, *m*/*z* = 447.15), afzelin (olive green, 17.037 min, *m*/*z* = 431.18), rhamnetin 5-rhamnoside (pink, 25.229 min, *m*/*z* = 461.15), and rhamnocitrin (orange, 29.953 min, *m*/*z* = 445.18).

Four flavonol rhamnosides, quercitrin, afzelin, rhamnetin 3-rhamnoside, and rhamnocitrin 3-rhamnoside, from LTE were measured at a wavelength of 245 nm and detected at approximately 14.3, 17.0, 25.2, and 29.9 min, respectively (Figure 1a). Their molecular weights, 447.15, 431.18, 461.15, and 445.18 *m*/*z* were detected in negative mode, respectively (Figure 1b,c).

### 3.3. Network of Active Molecules and Potential Target Genes

**Figure 2:**
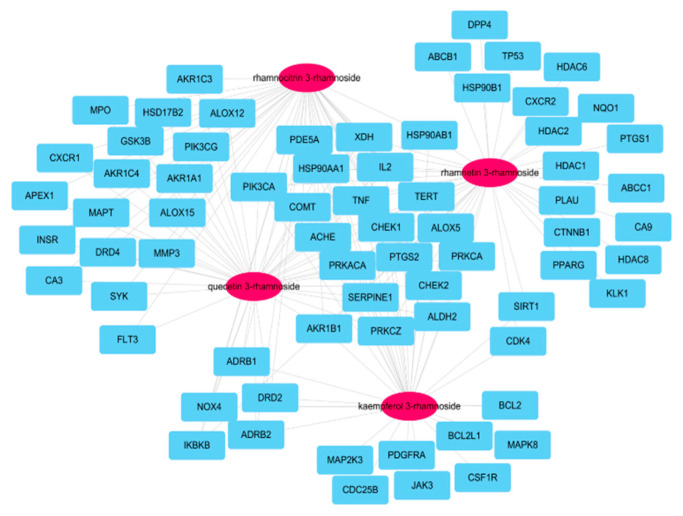
Network of four molecules (pink oval) and 70 potential target genes (cyan rectangles); this network consisted of 74 nodes and 160 edges.

A total of 173 genes was searched for linking to the four molecules according to SwissTargetPrediction database with ‘*Homo sapiens*’ species (Supplementary Materials Table S1). After searching for genes associated with COPD within GeneCards DB (Supplementary Materials Table S2), 70 potential target genes were identified by their intersection with genes related to four active molecules (Figure 2). Among the 173 genes, 20 were associated with the four main molecules: ACHE, AKR1B1, ALDH2, ALOX5, CHEK1, CHEK2, COMT, HSP90AA1, HSP90AB1, IL2, PDE5A, PIK3CA, PRKACA, PRKCA, PRKCZ, PYGS2, SERPINE1, TERT, TNF, and XDH.

### 3.4. Protein–Protein Interaction (PPI)

**Figure 3:**
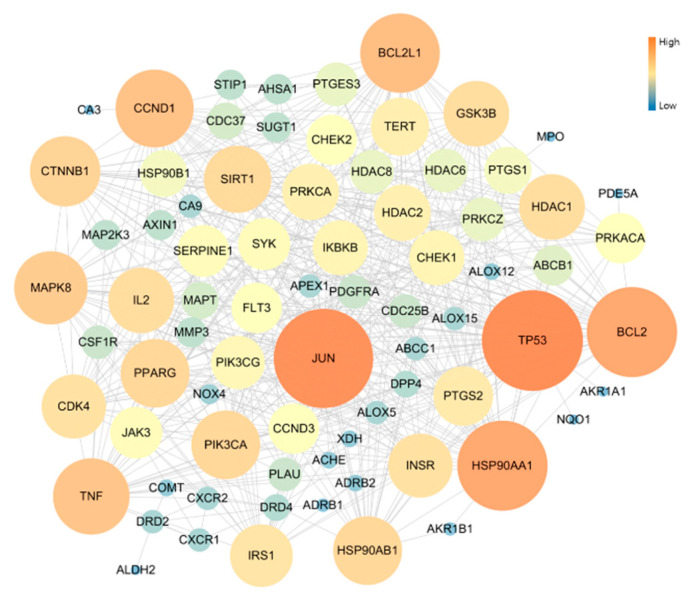
Topology analysis of PPI related COPD disease: the higher degree genes are expressed as large circles and in orange color.

The PPI network of 70 Potential COPD-related genes was constructed with 78 nodes and 507 edges. As result of network topology analysis, TP53, JUN, HSP90AA1, BCL2, BCL2L1, CCND1, TNF, MAPK8, CTNNB1, PIK3CA presented a high degree of interation. These were the top 10 genes (Figure 3).

### 3.5. Signal Pathway Analysis

**Figure 4:**
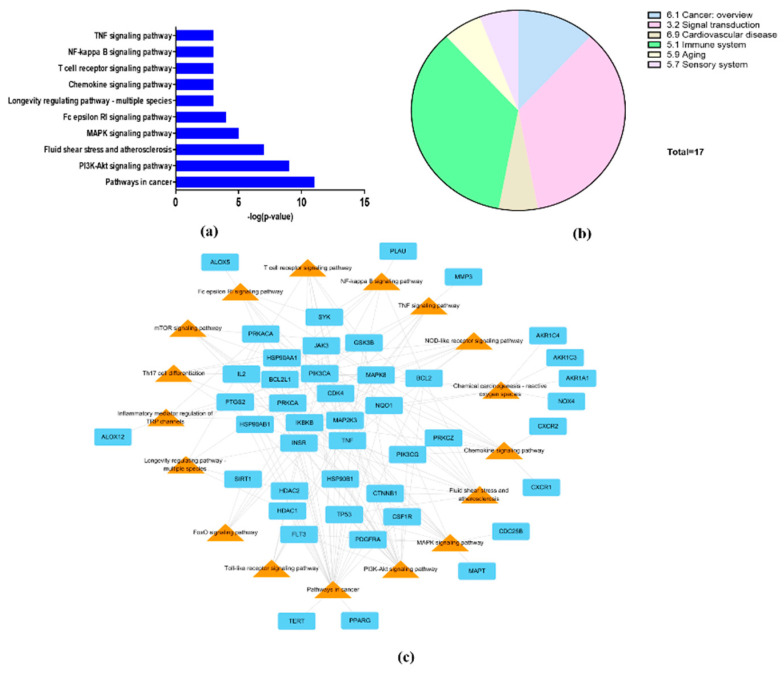
Network pharmacological analysis of LTE. (**a**) Top 10 KEGG pathways related to COPD with *p*-value, (**b**) group of COPD-related KEGG pathways; signal transduction and immune system were potential COPD-related pathways. (**c**) Network consisting of potential target genes (cyan rectangle) and KEGG pathways (orange triangles) with 61 nodes and 155 edges.

To explore the mechanism of the effect of *L. tanakae* on COPD, we analyzed KEGG pathways (*p* < 0.05). The top 10 COPD-related pathways were associated with cancer, PI3K-Akt signaling, fluid shear stress and atherosclerosis, MAPL signaling, Fc epsilon RI signaling, longevity regulation, chemokine signaling, NF-κB signaling, TNF signaling, and T cell receptor signaling (Figure 4a). The COPD-related pathways were classified to be associated with cancer overview, signal transduction, cardiovascular disease, immune system, aging, and sensory systems. Signal transduction and immune system were also potential pathways (Figure 4b). The genes associated with more than 10 pathways were IKBKB, MAPK8, PIK3CA, PRKACA, PRKCA, and TNF (Figure 4c).

### 3.6. Effects of LTE on the Generation of Inflammatory Cytokines in CSC Exposed H292 Cells

**Figure 5:**
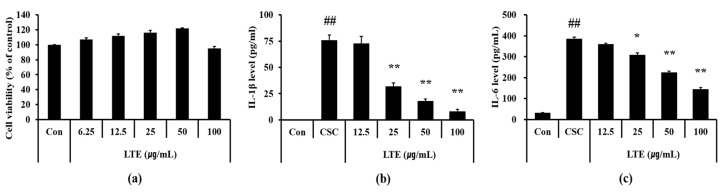
Effects of LTE on the production of inflammatory cytokines in CSC stimulated H292 cells. (**a**) cell viability, (**b**) IL-1β level, and (**c**) IL-6 level. This experiment was performed in triplicate. Data shown as the mean ± SD. ^##^, vs. Control, *p* < 0.01, *, **, vs. CSC stimulated cells, *p* < 0.05 and <0.01, respectively.

Based on the results of cell viability, we determined 100 μg/mL as a high concentration of LTE that resulted in a non-toxic effect (Figure 5a). CSC exposed H292 cells showed the marked elevation of IL-1β (Figure 5b) and IL-6 (Figure 5c) compared with non-treated cells. However, LTE treatment significantly declined the production of IL-1β and IL-6 in CSC exposed H292 cells, in a concentration dependent manner.

### 3.7. Effects of LTE on the Expression of Nrf2 and NF-κB in CSC Exposed H292 Cells

**Figure 6:**
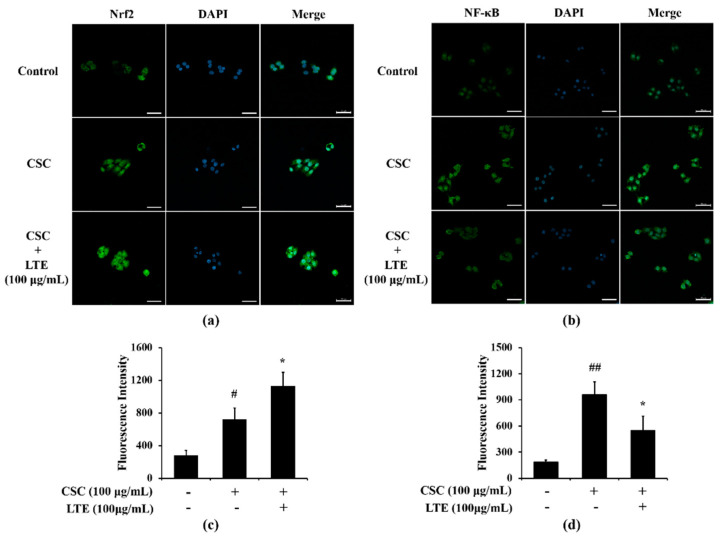
Effects of LTE on Nrf2 and NF-κB expression in CSC stimulated H292 cells. (**a**) representative figure for Nrf2, (**b**) representative figure for NF-κB, (**c**) quantitative analysis of Nrf2 expression, (**d**) quantitative analysis of NF-κB. The treatment of LTE increased Nrf2 translocation into the nucleus but decreased NF-κB translocation into the nucleus in CSC stimulated H292 cells. This experiment was performed in triplicate. Scale bar = 50 μm. Data shown as the mean ± SD. ^#^, ^##^, vs. Control, *p* < 0.05 and <0.01, respectively, *, vs. CSC-treated cells, *p* < 0.05.

A greater translocation of Nrf2 into nuclei was observed in CSC exposed H292 cells in comparison to that in non-treated cells (Figure 6a,c). LTE treatment further increased Nrf2 translocation into the nucleus in CSC exposed H292 cells. The translocation of NF-κB also increased into the nuclei of CSC exposed H292 cells compared with that in non-treated cells (Figure 6b,d). However, LTE treatment noticeably decreased the translocation of NF-κB into nuclei when induced by CSC.

### 3.8. Effects of LTE and Its Components on ROS Production

The scavenging DPPH and ABTS activities of the extracts and four ingredients were presented in Table 1. Antioxidant effect for DPPH and ABTS of LTE were 124.37 and 226.1 μg/mL, respectively. Additionally, rhamnetin 3-ramnoside and quercitrin showed antioxidant effects, but rhamnocitrin 3-rhamnoside and afzelin showed no antioxidant effect in DPPH and ABTS assay.

**Figure 7:**
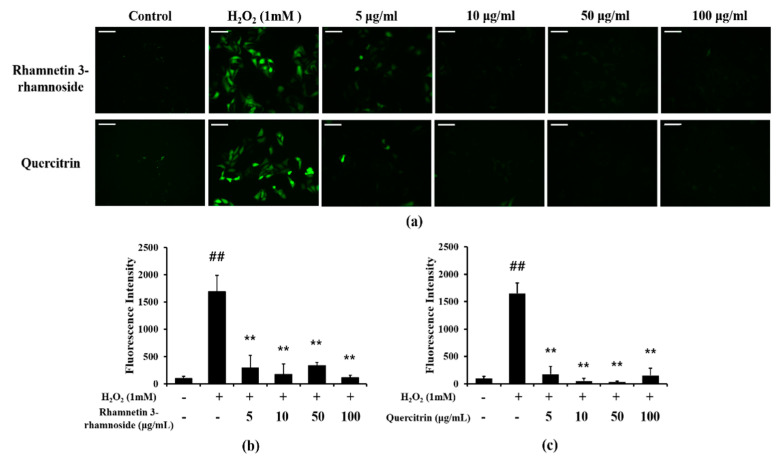
Effects of rhamnetin 3-ramnoside and quercitrin on ROS production. (**a**) representative figure of DCFDA stained HeLa cells, (**b**) quantitative analysis of ROS production in rhamnetin 3-rhamnoside-treated cells, (**c**) quantitative analysis of ROS production in quercitrin-treated cells. rhamnetin 3-ramnoside and quercitrin-treated cells exhibited marked reduction in ROS production induced by H_2_O_2_ treatment. This experiment was performed in triplicate. Scale bar = 50 μm. Data shown as the mean ± SD. ^##^, vs. Control, *p* < 0.01, **, vs. H_2_O_2_-treated cells, *p* < 0.01.

Additionally, we evaluated the effects of rhamnetin 3-ramnoside and quercitrin on H_2_O_2_-treated HeLa cells. H_2_O_2_-treated cells showed excessive ROS production, but rhamnetin 3-ramnoside and quercitrin-treated cells exhibited the significant reduction in ROS production compared with H_2_O_2_-treated cells (Figure 7a–c).

### 3.9. Effects of LTE on Inflammatory Indexes in CSC+LPS Exposed Mice

**Figure 8:**
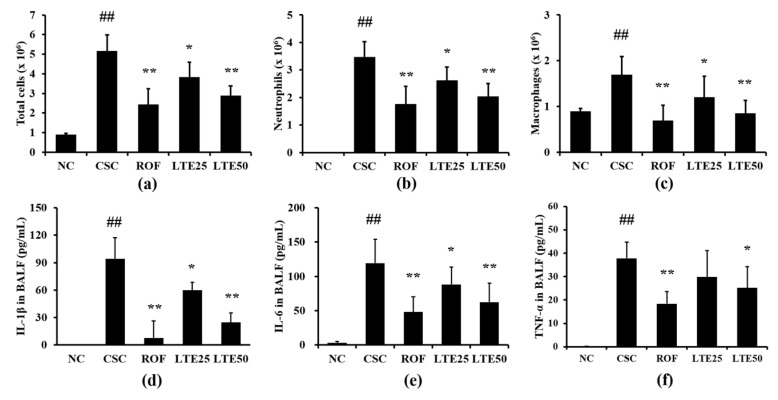
Effects of LTE on pathophysiological factors in CSC+LPS exposed mice. (**a**) Total cells in BALF, (**b**) neutrophils in BALF, (**c**) macrophages in BALF, (**d**) IL-1β levels in BALF, (**e**) IL-6 levels in BALF, and (**f**) TNF-α levels in BALF. ^##^, vs. NC, *p* < 0.01, *, **, vs. CSC+LPS, *p* < 0.05 and <0.01, respectively.

CSC+LPS exposed mice showed markedly elevated inflammatory cell counts for neutrophils, macrophages and total cells in BALF compared with those in the control group (Figure 8a–c, respectively). Roflumilast-treated mice exhibited the significant reduction in the inflammatory cell counts in BALF compared with CSC+LPS exposed mice. The administration of LTE significantly reduced inflammatory cell counts in BALF compared with those in CSC+LPS exposed mice, which was observed in dose-dependently manner. The levels of IL-1β, IL-6, and TNF-α in BALF were significantly elevated in CSC+LPS exposed mice than those in the control group (Figure 8d–f, respectively). Roflumilast-treated mice exhibited the significant reduction in IL-1β, IL-6, and TNF-α in BALF compared with CSC+LPS exposed mice. The administration of LTE reduced the levels of IL-1β, IL-6, and TNF-α in BALF in comparison with those in CSC+LPS exposed mice, which were noticeably observed in the group with a high dose of LTE. Although there is no significant difference between ROF group and LTE groups, ROF group slightly more decreased inflammatory cell count and cytokines than LTE groups.

### 3.10. Effects of LTE on Pathophysiological Alteration of Lung Tissue in CSC+LPS Exposed Mice

**Figure 9:**
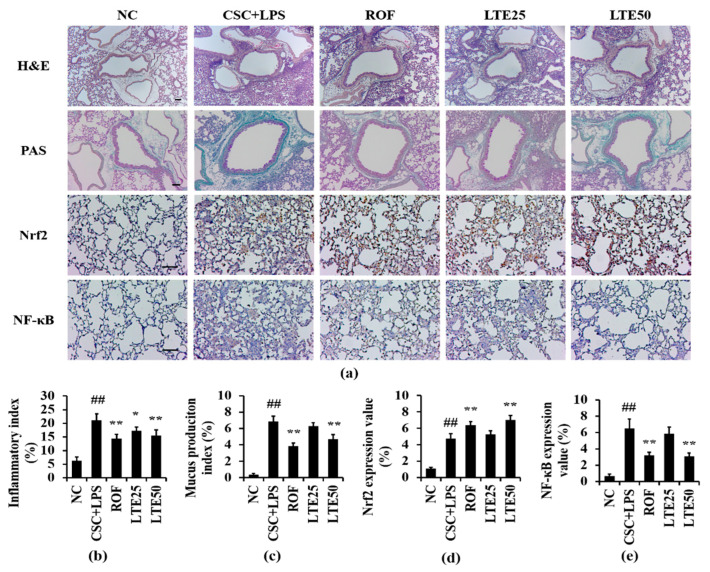
Effects of LTE on histological alteration in CSC+LPS exposed mice. (**a**) representative figure for H&E, PAS and ICH. (**b**) inflammatory index, (**c**) mucus production index, (**d**) Nrf2 expression value, (**e**) NF-κB expression value. Pulmonary inflammation and mucus production of lung tissue were determined using hematoxylin and eosin and PAS staining, respectively. The expression of Nrf2 and NF-κB on lung tissue was determined using immunohistochemistry. Scale bar = 100 μm. ^##^, vs. NC, *p* < 0.01, *, **, vs. CSC+LPS, *p* < 0.05 and <0.01, respectively.

CSC+LPS exposure to mice induced the recruitment of inflammatory cells and mucus production into lung tissue. Roflumilast-treated mice showed the marked reduction in inflammatory response and mucus production compared with the CSC+LPS exposed mice. The LTE treatment reduced the inflammatory response and mucus production caused by CSC+LPS exposure (Figure 9a–c). Although there is no significant difference between ROF group and LTE groups, ROF group slightly more decreased mucus production than LTE groups. The expression of Nrf2 on lung tissue increased to a greater extent in Roflumilast and LTE-treated mice with CSC+LPS exposure than that in CSC+LPS exposed mice (Figure 9a,d). In contrast, the administration of Roflumilast and LTE clearly reduced the expression of NF-κB on lung tissue owing to CSC+LPS exposure (Figure 9a,e).

## 4. Discussion

COPD is an important pulmonary disease with a high incidence and mortality [2]. Researchers have developed numerous therapeutic agents to treat COPD. However, candidates for controlling COPD have limited use owing to their low efficacy or toxicity. In this study, we analyzed the ingredients in LTE by HPLC and evaluated the therapeutic effects of LTE on CSC induced pulmonary inflammation using in vitro and in vivo experiments. In addition, the mechanisms of action of LTE on CSC induced airway inflammation were determined by network pharmacological analysis. HPLC revealed that the main components of LTE were quercitrin, afzelin, rhamnetin 3-rhamnoside, and rhamnocitrin 3-rhamnoside. In the results of in vivo and in vitro experiments, LTE showed ROS scavenging activity in DPPH and ABTS assays and its ingredients including rhamnetin 3-rhamnoside and quercitrin markedly decreased the ROS production induced by H_2_O_2_ treatment. Additionally, LTE significantly diminished inflammatory cytokines caused by the exposure to CSC+LPS or only CSC, accompanied by a reduction in inflammatory responses on lung tissue. LTE showed an elevation of Nrf2 and a decrease in NF-κB in both CSC exposed cells and CSC+LPS in the mice model. These results were consistent with those of network pharmacological analysis.

Result of network pharmacology analysis, total 17 effective COPD-related signaling pathways were analyzed as follows: pathways in cancer, PI3K-Akt, Fluid shear stress and atherosclerosis, MAPK, Fc epsilon RI, longevity regulating pathway-multi species, chemokine, T cell receptor, NF-κB, TNF, Inflammatory mediator regulation of TRP channels, Th 17 cell differentiation, chemical carcinogenesis-reactive oxygen species, NOD-like, mTOR, Toll-like and FoxO signaling pathways. A total 70 COPD–related genes were selected from four ingredients, of which 44 genes were involved in the COPD pathways. In particular, NF-κB, TNF, FoxO and PI3K-Akt are closely related with pathophysiological alteration induced by ROS. Exposure to irritant induces the activation of NF-κB and TNF signaling resulting the progression and aggravation of inflammatory responses on damaged lesions [29,30]. By contrast, FoxO and PI3K-Akt induce the enhancement of antioxidant system such as superoxide dismutase, catalase, Nrf2 and HO-1 in organism to reduce damage induced by ROS [31,32]. Therefore, the therapeutic effects of LTE on COPD is considered to be related with the reduction in damage caused by ROS.

CS is considered to be the most important factor in the progression of COPD owing to its many harmful constituents. CS induces the accumulation of inflammatory cells such as neutrophils and macrophages into lung tissue, which are associated with the alteration of the histology of lung tissue [4]. The neutrophils and macrophages involved in the generation of inflammatory mediators, including ROS, cytokines, and chemokines, result in the exacerbation of inflammatory responses [6]. In addition, neutrophils produce the protease that destroys normal lung tissue via the degradation of connective tissues [33]. In this study, LTE treatment reduced the number of inflammatory cells, including neutrophils and macrophages in the BALF of CSC exposed mice, which was accompanied by a decline of the inflammatory response of lung tissue. Furthermore, LTE decreased the production of TNF-α, IL-6, and IL-1β in CSC exposed cells and in CSC+LPS exposed mice. These results indicate that LTE effectively suppressed pulmonary inflammation induced by CSC exposure.

Inflammatory responses are involved in various signaling pathways. In particular, NF-κB is intimately linked with the progression of inflammatory response [2]. NF-κB can be activated by multiple stimuli, such as air pollutants, allergens, fine dust, bacteria, toxins, and chemicals in the airway [34]. Activated NF-κB translocates into the nucleus, which switches on various inflammatory genes, resulting in the amplification of inflammation on damaged lesions [10]. During the development of COPD, CS produces ROS, which stimulates the activation of NF-κB [6]. Therefore, the suppression of NF-κB is regarded to be a critical treatment strategy for controlling COPD. In this study, LTE effectively inhibited the activation of NF-κB in CSC+LPS exposed mice, which led to a reduction in inflammatory cytokines. These results indicate that administration of LTE suppressed pulmonary inflammation induced by CSC+LPS exposure via the inhibition of NF-κB.

Exposure to CS also induces oxidative damage to an organism when ROS is produced. Healthy organisms protect against oxidative damage through the enhancement of antioxidant systems, such as reduced glutathione, glutathione reductase, superoxide dismutase, and catalase production [35]. However, repeated exposure to CS continuously produces excessive ROS, which eventually causes an imbalance between ROS and the antioxidant system, leading to a pathophysiological alteration of the respiratory tract [36]. Therefore, the enhancement of the antioxidant system is an aspect for controlling COPD. Nrf2 is an antioxidant transcription factor triggered by oxidative stress induced by various stimuli [35], and binds to Kelch-like ECH associated protein1 (Keap1) under homeostatic condition. However, under oxidative conditions, Nrf2 released from Keap1, translocates into nuclei and binds to antioxidant response elements, leading to the transcription of several genes, such as hemeoxygenase-1 and nicotinamide adenine dinucleotide phosphate (NAD(P)H) quinone oxidoreductase 1 [37]. These events eventually protect organisms against oxidative stress via the enhancement of their antioxidant statuses. Nrf2 is also involved in the progression of inflammation as it suppresses the activation of NF-κB by reducing intracellular ROS levels and reducing the degradation of IκB-α and translocation of NF-κB into nuclei, leading to a decrease in the generation of inflammatory cytokines, including TNF-α and IL-1β [35,38,39]. In this study, LTE induced the translocation of Nrf2 into the nucleus, with a reduction in NF-κB translocation into the nucleus in CSC exposed cells and CSC+LPS exposed mice. This eventually decreased inflammatory cytokines and inflammatory responses in the lung tissues of CSC+LPS exposed mice. Therefore, the therapeutic effects of LTE against CSC+LPS caused pulmonary inflammation were intimately linked with the modulation of Nrf2 and NF-κB signaling.

Therapeutic effects of the components of LTE on CSC induced pulmonary inflammation occurred owing to its various pharmacological properties, e.g., anti-inflammatory and anti-oxidative properties.

We detected quercitrin, afzelin, rhamnetin 3-rhamnoside, and rhamnocitrin 3-rhamnoside in LTE via HPLC analysis. Quercitrin decreased oxidative stress and inflammation induced by various stimuli in cells by suppressing NF-κB activation and Nrf2 translocation into the nucleus [40,41]. Afzelin has exhibited anti-inflammatory and anti-oxidative properties in several studies [34,42,43,44], agreeing with our results

## 5. Conclusions

Collectively, our study provides evidence that LTE inhibited inflammatory responses in CSC+LPS-exposed mice in vivo and CSC-exposed H292 cells in vitro. These effects may be involved in the activation of Nrf2 and suppression of NF-κB. Therefore, we considered that LTE has potential as a therapeutic agent for controlling pulmonary inflammation induced by CS.

## Figures and Tables

**Table 1:** IC_50_ values in antioxidant assays of LTE and its ingredients.

Sample	DPPH IC_50_ (μM)	ABTS IC_50_ (μM)
Gallic acid (positive control)	20.43	13.81
LTE (μg/mL)	124.37	226.1
Rhamnetin 3-ramnoside	17.90	36.36
Rhamnocitrin 3-rhamnoside	-	-
Afzelin	-	-
Quercitrin	23.72	64.21

## Data Availability

Data are contained within the article and supplementary material.

## Supplementary Materials

The following supporting information can be downloaded at: https://www.mdpi.com/article/10.3390/antiox11101885/s1, Figure S1. Purity information of four single compounds by HPLC chromatogram and extracted-ion chromatogram (XIC). Table S1: Information of small molecules and genes in *Loranthus tanakae*; Table S2: COPD-related genes in Gene Cards database.
