# Supplementary material for: Loranthus tanakae Franch. & Sav. Suppresses Inflammatory Response in Cigarette Smoke Condensate Exposed Bronchial Epithelial Cells and Mice
Source: Antioxidants (Basel). 2022 Sep 23;11(10):1885. doi: 10.3390/antiox11101885 (PMC9598098; doi:10.3390/antiox11101885)
Supplement: Supplementary file 1 [file antioxidants-11-01885-s001.zip › supplementary Materials Figure S1.pdf]

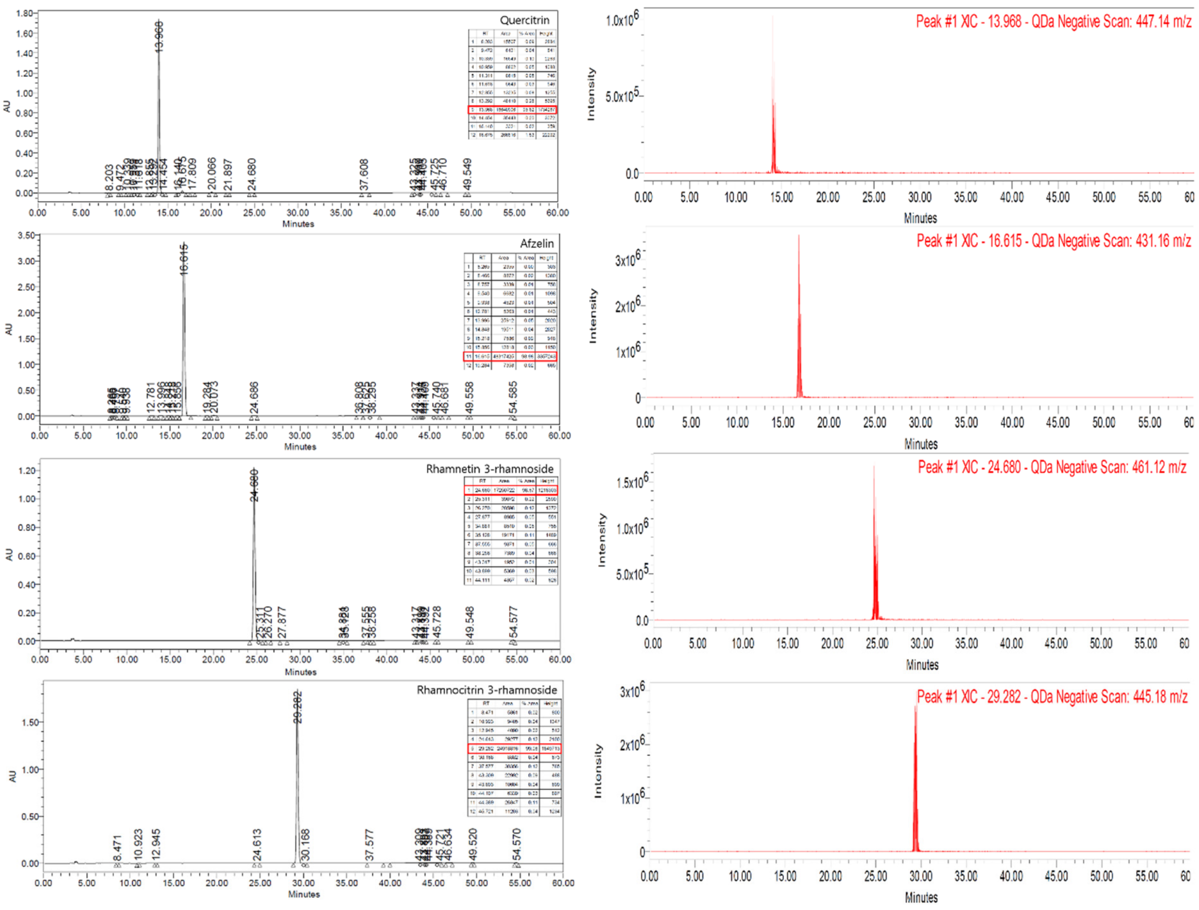

Supplementary Material Figure S1. Purity information of four single compounds by HPLC chromatogram and extracted-ion chromatogram (XIC).
